# Assessing the Impact of Religion and College Life on Consumption Patterns of Ultra-Processed Foods by Young Adults: A Cross-Sectional Study

**DOI:** 10.3390/nu16111619

**Published:** 2024-05-25

**Authors:** Silvia Navarro-Prado, Jacqueline Schmidt-RioValle, Ángel Fernández-Aparicio, Miguel Ángel Montero-Alonso, Javier S. Perona, Emilio González-Jiménez

**Affiliations:** 1 Department of Nursing, Faculty of Health Sciences, Melilla Campus, University of Granada, 52005 Melilla, Spain; silnado@ugr.es; 2 Department of Nursing, Faculty of Health Sciences, University of Granada, 18016 Granada, Spain; jschmidt@ugr.es (J.S.-R.); emigoji@ugr.es (E.G.-J.); 3 Instituto de Investigación Biosanitaria (ibs.GRANADA), 18014 Granada, Spain; 4 Department of Statistics and O.I., Faculty of Medicine, University of Granada, 18016 Granada, Spain; mmontero@ugr.es; 5 Department of Food and Health, Instituto de la Grasa-CSIC, Campus of the University Pablo de Olavide, 41013 Seville, Spain; perona@ig.csic.es

**Keywords:** ultra-processed foods, university students, eating patterns, healthy habits, health promotion

## Abstract

(1) Background: University students, often constrained by time and influenced by socio-economic factors such as culture and religion, frequently adopt diets centred on ultra-processed foods (UPFs), increasing the risk of long-term non-communicable diseases. This study aimed to assess UPF consumption among Spanish university students and explore the potential impact of religion and the academic year on their eating habits. (2) Methods: In a cross-sectional study of 257 university students aged 18–31, UPF consumption was assessed using NOVA food classification at the academic year’s start and end. Chi-square and Wilcoxon tests analysed UPF consumption changes, while binary logistic regression identified associations between religion and weekly UPF consumption. (3) Results: Muslim students had a consumption of industrial bakery products almost five times [95% CI: 2.694–9.259] higher than that observed among Christians. Similar data were observed for artificial juice consumption (OR = 3.897, 95% CI = 2.291–6.627) and candy consumption (OR = 3.724, 95% CI = 2.051–6.762). Moreover, a greater percentage of calories and grams of saturated fats from UPFs was observed for Muslims at the end of the study. (4) Conclusions: Highlighting the impact of religion on UPF consumption among students underscores the necessity of monitoring and intervening in dietary habits to prevent undesirable long-term complications such as cardiovascular diseases.

## 1. Introduction

University students represent a population group exposed to changes and risks typical of this transitional stage between adolescence and middle adulthood [1,2]. In some cases, greater autonomy and independence from the family core imply the need to make decisions about which foods to consume on a daily basis, making the university stage a key period for the adoption of lasting nutritional habits [3,4].

The university is an environment in which the adoption of healthy habits has not traditionally been promoted in the student community. Over the last decade, some universities have implemented actions that reflect their commitment to the necessary promotion of the health, well-being and quality of life of their community, although in some cases paying little attention to the nutritional habits of the students [5]. In this context, universities should place larger emphasis on promoting healthy nutritional habits, especially considering the sensitivity of university students to health promotion and prevention activities, since their lifestyles greatly affect their physical, psychological and mental development [6,7].

In general, the limited time that university students spend planning their daily diet, coupled with issues of economy and ease of access, leads to choosing diets based mainly on ready-made or quick-to-prepare meals prepared using ultra-processed products [8,9]. Different studies have shown that ultra-processed products are characterised by low amounts of fibre, protein and micronutrients while being energy-dense and high in saturated and trans fatty acids, added sugars and sodium [10,11]. These characteristics of foods may favour the development of non-communicable diseases (NCDs), such as type-2 diabetes mellitus, metabolic syndrome, obesity, cardiovascular disease and cancer [12]. In this line, lower adherence to the Mediterranean diet has been reported in university students consuming these ultra-processed foods [13]. In particular, higher levels of blood pressure (BP) [14] and of waist circumference [13] were observed among male university students consuming this type of product.

Although no studies have explored the influence of religion on the dietary patterns of ultra-processed foods (UPFs) among university students to date, scientific evidence suggests that culture and/or religion may act as modulating factors for the adoption of nutritional habits among young adults [15]. In fact, different studies performed in diverse countries around the world have analysed the consumption of UPFs among university students. In Spain, according to Latasa et al. [16], between 1990 and 2010, the purchases of UPFs almost tripled from 11.0% to 31.7%. Mwafi et al. [17] found that 59.4% of Muslim students at Mutah University in southern Jordan consumed UPFs two or more times per week. They also observed significant correlations between fast food consumption and that of chips, processed meat, coffee and sweets. However, they did not find a significant relationship between fast food consumption and students’ religion. Aravena et al. [18], in their study of university students in Valparaíso (Chile), found a high consumption of sauces and fat spreads, sausages and other processed meat products, together with industrial bakery products. Likewise, Mamani-Urrutia et al. [19] found a high weekly consumption of snacks, biscuits, sweetened milk drinks and packaged juices in university students in the city of Lima (Peru). These findings highlight the serious public health problem that the consumption of ultra-processed products represents, particularly among university students.

The university stage is a crucial phase in which university students are consolidating their lifestyles and nutritional habits [6,7]. As already stated, different socioeconomic factors such as culture and the university context could potentially influence the adoption of nutritional habits in university students. Additionally, some studies underscore that the constrained time available to university students for meal planning, coupled with their newfound independence and autonomy, may lead them to opt for highly processed food choices. However, to the best of our knowledge, the consumption of ultra-processed products among Spanish university students belonging to diverse cultures and religions has not been explored in Spain. Melilla, a Spanish university city situated in North Africa, with a significant Christian and Muslim student community, provides a unique opportunity to investigate not only whether this cultural and religious diversity positively impacts the dietary preferences of students but also whether the course of the academic year influences the dietary styles of university students. Moreover, the analysis of these factors could guide the direction of cardiovascular disease prevention strategies, acting early in a young adult population susceptible to changes in their lifestyles. Consequently, this study aimed to assess the consumption of ultra-processed products among a sample of Spanish university students, investigating the potential influence of religion and the course of the university academic year on their consumption patterns.

## 2. Materials and Methods

### 2.1. Study Design and Subjects

A cross-sectional study with two assessment moments (at the beginning and at the end of the academic year) was carried out on university students. A total population of 257 university students were selected by random sampling among all of the 1188 students enrolled at the university campus in the city of Melilla, a Spanish city located on the north-west coast of Africa. Melilla is a culturally rich city, characterized by the harmonious coexistence of diverse ethnic groups and cultures.

### 2.2. Data Collection

**Figure 1:**
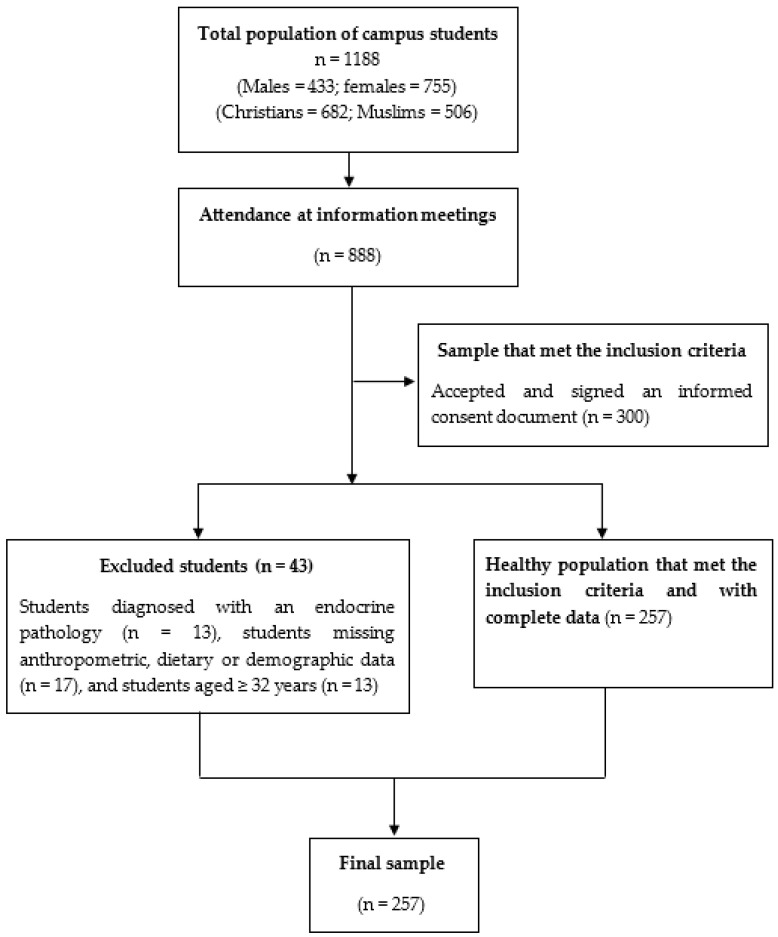
A flow diagram of the recruitment process.

The Melilla campus consists of three university centres: the Faculty of Educational and Sport Sciences, the Faculty of Social Sciences and Law and the Faculty of Health Sciences. Therefore, an inclusion criterion for our study was to be enrolled in a degree programme offered by any of these three centres. The students were required to sign an informed consent document to participate in the study. Students with a prior diagnosis of endocrine or metabolic diseases, as well as students who refused to participate in the study, were excluded from the study. Figure 1 summarizes the participant recruitment process, which shows that among the 1188 participants, 257 finally participated in the study after the application of the inclusion and exclusion criteria.

Informational meetings were conducted for the 1188 students enrolled at the university campus of Melilla, with 888 students attending all of them. At these meetings, participants were informed about the different assessments and questionnaires that they would need to complete if they finally decided to participate in the study. An informed consent document describing the study in detail was provided to all attendees. At these meetings, the aforementioned inclusion criteria were applied, and a total of 300 students were selected. Of these students, 43 were excluded for different reasons: being diagnosed with an endocrine pathology (n = 13); incomplete anthropometric, dietary or demographic data (n = 17); and age ≥ 32 years (n = 13). All participants who met the inclusion criteria and provided complete data were included in our study. Consequently, 257 young university students eventually participated in our study.

The Ministry of Education and Youth of the Government of Melilla approved the study. Furthermore, the Ethics Committee of the University of Granada (Code 841) approved the study and the informed consent document. All of the participants signed the informed consent document, and the confidentiality of the participants’ information was guaranteed by coding the data. This research was performed in strict compliance with the international code of medical ethics established by the World Medical Association and the Declaration of Helsinki.

### 2.3. Dietary Intake

A validated food frequency questionnaire (FFQ) consisting of 168 food items was completed by each participant to assess typical dietary intake over the past 12 months [20]. The participants were asked to report the frequency of consumption of each food item per day, week or month.

Furthermore, the subjects were asked to complete a 72 h food record (i.e., Thursday, Friday and Saturday) to capture weekly variations across weekdays and the weekend. The use of a 72 h food record as a valid method to assess nutrient intake is supported by the literature because the record collects accurate data on the typical or average diet [21]. The 72 h food records were conducted by trained investigators in a face-to-face interview, where individuals were asked to recall all food consumed in the preceding 72 h, including nutritional supplements and beverages. Standard household measures and pictorial food models were employed during the interviews to define amounts when requested. When researchers suspected that a product added to the 72 h food record might have a high degree of processing, participants were asked to indicate the brand of the food or whether it was packaged. The nutritional computer application Diet Source^®^ version 3.0 was used to analyse the nutritional information (% of calories and amounts of macronutrients and micronutrients).

We applied the NOVA food classification and categorized each food and beverage item from the FFQ and the 72 h food record into 1 of the 4 food groups based on their extent and purpose of industrial food processing [22]: (1) unprocessed/minimally processed foods are fresh, frozen, ground, pasteurized or (nonalcoholic) fermented after separation from nature (e.g., fruit, vegetable, milk, meat, legumes); (2) processed culinary ingredients are substances extracted from foods and used in common culinary preparation, cooking and seasoning of group 1 foods (e.g., table salt, sugar, vegetable oils and butter); (3) processed foods are made by adding salt, sugar or other group 2 ingredients to group 1 foods (e.g., canned vegetables in brine, canned fish, freshly made breads and cheeses); and (4) UPFs are food and drink formulations of multiple substances, mostly of exclusive industrial use (e.g., high-fructose corn syrup), and they are manufactured through a series of complex industrial processes (e.g., hydrogenation) and often contain cosmetic food additives (e.g., colours, flavours, emulsifiers) that disguise any undesirable sensorial properties of the final product. Some examples are sugary drinks, industrial-processed packaged breads with added emulsifiers and preprepared frozen or shelf-stable meals made with modified starches, stabilizers or flavour enhancers. In regard to the 72 h food record, after classifying each food into 1 of the food groups of the NOVA classification, the calories and key nutrient composition of UPFs that had been ingested by each participant were obtained using the nutritional computer application Diet Source^®^ version 3.0.

### 2.4. Anthropometric Measurements

At the beginning of the academic year, anthropometric parameters, including height, weight and body mass index (BMI), were assessed following the guidelines of the International Society for the Advancement of Kinanthropometry [23]. A self-calibrating Seca^®^ 861 class (III) digital floor scale, which had a precision up to 100 g, was used to measure the weights of the participants. The height of each participant was measured using a Seca^®^ 214* portable stadiometer using the following procedure: the participant was asked to stand in an upright position with his/her back and heels against the stadiometer and his/her head oriented in the Frankfurt plane. Then, the horizontal headpiece was placed on the top of their head. BMI was calculated by dividing the participant’s weight by the square of his/her height (kg/m^2^). The same trained research assistant performed all the measurements.

### 2.5. Blood Pressure

A previously calibrated aneroid sphygmomanometer and a Littmann^®^ stethoscope (3M Spain S.L., Madrid, Spain) were used to measure BP at the beginning of the academic year. The recommendations for BP measurement proposed by the Subcommittee of Professional and Public Education of the American Heart Association Council on High Blood Pressure Research were followed [24]. Each participant was required to avoid eating, drinking alcohol or caffeine, smoking, exercising and bathing for at least 30 min before a BP measurement. BP was measured with the subject seated on a chair for 5 min with his/her back supported, feet flat on the floor and wrist relaxed at heart level. The results were interpreted according to the Korotkoff sounds: phase I, systolic BP; and phase V, diastolic BP [24].

### 2.6. Other Variables

Another variable studied was religion, and each student self-identified the religion that he/she practised (Islam or Christianity). The Religious Attitudes Questionnaire, which was developed and validated by Elzo Imaz et al. [25], was used to measure this variable.

### 2.7. Statistical Analysis

A statistical analysis was carried out to compare Christians and Muslims in various aspects. To report the physical and sociodemographic characteristics of participating students, continuous variables were presented as mean ± standard deviation, while categorical variables were expressed as frequency (percentage). The normality of quantitative variables was assessed using the Shapiro–Wilk test, determining the choice between the *t*-test/Welch’s *t*-test or Mann–Whitney U test accordingly. Qualitative variables underwent analysis using the Chi-square test or Fisher’s Exact test. The number of participants that consumed ultra-processed products at different time points was assessed using the Chi-square test or, where appropriate, Fisher’s Exact test and was reported as frequency (percentage). Given the dispersion of the students’ responses, the frequency of weekly consumption among Christian and Muslim students participating in the study was assessed by applying the Wilcoxon Ranks test for related samples and expressed as median and interquartile range. Data on total energy and ultra-processed foods energy and on the consumption of key nutrients from UPFs are reported as mean ± standard deviation, and the Wilcoxon Ranks test was applied for related samples. Odds ratios were calculated through binary logistic regression analysis, with Christians as the reference, and they were adjusted for religion, and for sex and religion, with 95% confidence intervals (CI). Food assessments (low vs. high intake) were evaluated and estimated through using the recommended intakes proposed by the World Health Organization (WHO) [26]. Statistical analyses were performed using SPSS v25.0 [27], and significance was set at *p* < 0.05.

## 3. Results

Of the 257 students that participated in the study, 141 self-reported as Christian (51 men and 90 women) and 116 as Muslim (37 men and 79 women). All of them belonged to the Campus of the University of Granada in the Autonomous City of Melilla.

### 3.1. Physical and Sociodemographic Characteristics of Participating Students

Table 1 shows the physical characteristics and nutritional status of the students, showing a higher proportion of overweight status among Christian and Muslim men (40.9%) compared to women (19.5%). Regarding blood pressure levels, up to 74.5% of Christian men were prehypertensive, followed by 70.3% of Muslim men. For the variable “place of residence” during the academic year, almost all Muslim male students resided in the family residence as opposed to Christian male students (*p* = 0.003), who lived independently of their families. Likewise, Muslim female students resided mostly in the family residence as opposed to Christian female students (*p* < 0.001). Regarding the place where participants had lunch during the academic course, there were statistically significant differences between Muslim male (*p* = 0.015) and female (*p* < 0.001) students compared to Christian male and female students, respectively.

### 3.2. Consumption of Ultra-Processed Products According to Religious Group

Table 2 shows that in the first assessment, 50.4% of Christian students consumed artificial juices, a percentage that dropped to 47.5% in the second assessment, showing statistically significant differences (*p* = 0.046). Similarly, a reduction in the consumption of artificial juices was also observed in Muslim students, which was reduced from 77.6% in the first assessment to 72.4% in the second assessment (*p* = 0.014). In relation to the consumption of ultra-processed baked foods, a statistically significant decrease was observed in both groups between the first and second assessment. Among Christian students, the consumption of these products lowered by 23.2%, and among Muslim students by 8.0%. With regard to the consumption of candies, statistically significant differences were also observed, i.e., 61.0% of Christian students consumed sweets in the first assessment, a percentage that decreased to 51.1% during the second assessment (*p* < 0.001). Consumption among Muslim students was equally high, and the decrease in consumption was also statistically significant (*p* = 0.005), with 81% consuming during the first assessment and 74.1% in the second assessment.

### 3.3. Data on Energy and Key Nutrients Derived from Ingestion of Ultra-Processed Products

**Figure 2:**
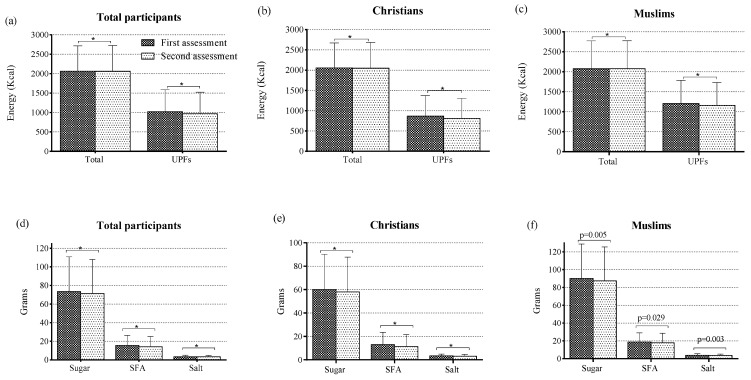
Data on total energy and ultra-processed food energy (**a**–**c**) and on the consumption of key nutrients from UPFs (**d**–**f**). UPFs, ultra-processed foods; SFA, saturated fatty acids. * *p* < 0.001.

Figure 2 shows the referenced data on total and UPF energy and key nutrients derived from the consumption of UPFs in the first and second assessments according to religion. At the beginning of the study, the daily energy intake (kcal) from ultra-processed products was 42.2% and 58.1% for Christians and Muslims, respectively, whereas at the end of the study, these intakes were 39.6% and 55.8% for Christians and Muslims, respectively. Statistical significance (*p* < 0.001) was observed for all data in these figures, except for saturated fatty acids (*p* = 0.029), at both the initial and final assessment points of the study, among both Christian and Muslim participants.

### 3.4. Frequency of Weekly Consumption of Ultra-Processed Products According to Religious Group

As shown in Table 3, among Christian students, there was a statistically significant decrease (*p* < 0.001) in the weekly consumption of industrial bakery foods and sausages from the first to the second assessment. This was true also for the frequency of weekly consumption of sugary drinks (*p* = 0.027), cereals (*p* = 0.020), sweets (*p* = 0.002) and chocolate (0.002). In Muslim students, a statistically significant decrease (*p* < 0.001) was observed in the frequency of weekly consumption of cereals and sweets between the first and second assessment, which was also observed for the frequency of weekly consumption of soft drinks (*p* = 0.007) and chocolate (*p* = 0.003).

### 3.5. Influence of Religious Group on Consumption of Ultra-Processed Products

We found no association between belonging to a Christian or Muslim religious group and the frequency of consumption of any of the ultra-processed products (Table 4). However, the number of students with a low consumption of industrial bakery products compared to those with a high consumption was 4.995 (95% confidence interval [CI] = 2.694, 9.259) times higher in Christian students than in Muslim students. The same was true for the consumption of artificial juice (OR = 3.897, 95% CI = 2.291, 6.627), candies (OR = 3.724, 95% CI = 2.051, 6.762) and chocolate (OR = 2.183, 95% CI = 1.301, 3.664).

## 4. Discussion

To the best of our knowledge, this is the first study assessing the possible influence of religion and university life on the consumption of UPFs in Christian and Muslim Spanish university students. The main findings presented in the present cross-sectional study are as follows: (i) the consumption of UPFs was high in both Muslim and Christian students; (ii) the frequency of weekly consumption of all UPF subgroups, especially industrial juices and sugary drinks, was higher in Muslim than in Christian students; and (iii) the intake of industrial pastries by Muslim students was five times greater than that of Christian students. These results suggest the need to improve eating patterns in a university context, taking into account the religious component.

Our results show a higher prevalence of overweight status among Christian and Muslim men compared to women. These results are similar to those found in other studies carried out in Spain, such as that of Rodríguez-Rodríguez et al. [28], who also described a higher prevalence of overweight status in men than in women. Furthermore, in a population of 312 Saudi university students, Abdel-Megeid et al. [29] reported an overweight rate of 21%, with a higher prevalence among the male students (23%). In our study, the different prevalence of overweight status observed in university students of both sexes could be related to different habits for men and women, making it necessary to investigate other potentially influential factors.

However, factors such as the place of residence during the academic year or the place where food is most frequently consumed (home or away) should be taken with caution considering that the Muslim population usually eats at home (more than 90% of students) as opposed to the Christian population who eat in different places. This fact might indicate that they maintain their eating habits during their time at university, including the regular consumption of UPFs, due to cultural reasons.

The higher prevalence of prehypertension among Christian and Muslim men than among women is also noteworthy. These results are consistent with the systematic review study developed by Del Alba Giménez et al. [30], who describes a prevalence of prehypertension in 28.9% of university men and in 11.1% of women. The prevalence of prehypertension observed in our study is of concern, particularly among Christian and Muslim males, considering the young age of the students. Several studies have shown that the prevalence of prehypertension is often higher in men than in women, which could be due to differences in hormonal activity during early life [31,32]. In addition, in our study it also coincides with a higher prevalence of overweight status. This situation deserves special attention given that these subjects might be more prone to develop hypertension in the future and, consequently, have a higher risk of cardiovascular events, regardless of other possible risk factors [33].

Regarding the frequency of consumption, both Christian and Muslim students showed a significant decrease in the weekly consumption of most ultra-processed products, including sugary drinks, cereals, sweets and chocolate between the first and second assessment (median frequency: 1–2 times a week). However, Christians but not Muslims reduced the consumption of ultra-processed bakery products and sausages. In Muslim students, a higher weekly frequency of consumption was observed for most ultra-processed products (median frequency of 2 times a week), which was very high for artificial juices and sugary beverages (4 and 5 times a week). These results are similar to those described in other studies conducted among university students in Saudi Arabia [34] and Lebanon [35], in which 74.5% and 58.7% of university students consumed ultra-processed products twice a week, respectively. Our data indicate that the consumption of UPFs is frequent among university students in Melilla.

This situation suggests that educational programmes on healthy eating developed in Spain such as the NAOS Strategy [36] have not had sufficient social impact to reduce the consumption of ultra-processed products. According to Mytton et al. [37], a 20% tax on ultra-processed products could be an effective social measure to dissuade people from consuming them. In Spain, only the Catalonia region has established a tax on the consumption of sugary drinks [38], but not on other ultra-processed products, and at the moment there are no conclusive results about the effectiveness of said tax.

On the other hand, Kabir et al. [39] described that the eating behaviour and dietary intake of young university students are influenced by individual, social, university-related and environmental factors. In Spain, a Royal Decree (126/2015, issued on February 27th) mandates that nutrition information that must be included on food labels, a matter also regulated by European Union Regulation No. 1169/2011. These legislations, complemented by other actions, could contribute significantly to increasing healthier eating patterns and habits, ultimately preventing the development of NCD [18]. In this context, the university setting represents an opportunity to implement institutional policies that favour the development of healthier university campuses in which the consumption of non-processed or minimally processed foods is promoted [40,41].

In our study, the values of saturated fatty acids ingested from UPFs at the beginning of the study by Muslim participants were practically the same as those reported at the end of the study. These findings align with the observation that Muslim students consumed five times as many ultra-processed products (such as industrial pastries) compared to Christian students. Among the UPFs studied, sausages, industrial pastries, chocolate and precooked foods were rich in saturated fatty acids. Since we found no significant differences in the consumption of precooked foods and since Muslims tend to eat much fewer sausages for religious reasons, the higher intake of saturated fatty acids should be attributed to the consumption of industrial pastries and chocolate. Therefore, Muslim students have a lower probability (0.20) of having a low consumption of ultra-processed products. Based on these results and considering that the majority of students lived in their family homes during the academic year, we can assume that the consumption of ultra-processed products is related to both family and cultural habits. These findings are clinically and socially relevant, highlighting the need to develop educational intervention programmes aimed at promoting healthy nutritional habits among the general population but also among university students from different cultures and religious beliefs and their immediate family environment [42].

The main limitation of this study stemmed from the potential bias introduced by the use of the food consumption frequency questionnaire and the 72 h recall questionnaire, as they could lead to alterations in participants’ usual dietary habits during the survey period.

## 5. Conclusions

In conclusion, both Muslim and Christian students presented a high consumption of UPFs. However, Muslim students consumed all subgroups of ultra-processed products more frequently than their Christian counterparts, with a much higher frequency of weekly consumption of industrial juices and sugary drinks. These results show that the diet of the university students could be improved to move closer to a healthy eating pattern, particularly among Muslim students. Concerning the potential impact of religion on the consumption of ultra-processed foods, we found that Muslim students had an intake of such foods nearly five times greater than Christian students. These findings highlight the importance of monitoring the consumption of ultra-processed products among young adults both in a clinical context and in health promotion interventions, with the purpose of preventing and managing alterations related to dietary habits, including metabolic syndrome. Since these diseases exhibit distinct trends based on gender, it is crucial to provide gender-specific management strategies, also considering cultural and religious influences, before the easy availability and popularity of UPFs make them a core component of young adult dietary patterns, which could undesirably lead to the occurrence of long-term complications such as cardiovascular diseases.

## Figures and Tables

**Table 1:** Physical and sociodemographic characteristics of participating students.

Variables	Males (n = 88)		Females (n = 169)	
	Christians (n = 51)	Muslims (n = 37)	Christians (n = 90)	Muslims (n = 79)
*Physical characteristics*				
Height (cm)	178.50 ± 7.13	178.24 ± 7.15	163.77 ± 5.74	165.67 ± 6.04
Weight (kg)	80.32 ± 12.52	75.35 ± 12.04	60.46 ± 9.85 *	62.10 ± 10.50
BMI (kg/m^2^)	25.19 ± 3.56 *	23.70 ± 3.28	22.54 ± 3.45	22.60 ± 3.54
Nutritional status				
Normal weight	29 (56.9%)	23 (62.2%)	71 (78.9%)	65 (82.3%)
Overweight	22 (43.1%)	14 (37.8%)	19 (21.1%)	14 (17.7%)
SBP (mmHg)	121.42 ± 14.30	123.11 ± 11.01	111.62 ± 11.35	112.95 ± 11.32
DBP (mmHg)	70.22 ± 13.20	72.05 ± 7.20	65.34 ± 7.90	67.18 ± 6.30
Blood pressure classification				
Normal	10 (19.6%)	10 (27.0%)	60 (66.7%)	48 (60.7%)
Prehypertensive	38 (74.5%)	26 (70.3%)	30 (33.3%)	30 (38.0%)
Hypertensive	3 (5.9%)	1 (2.7%)	0	1 (1.3%)
*Sociodemographic characteristics*				
Age (year)	22.27 ± 4.36	20.97 ± 2.72	23.30 ± 6.01	21.42 ± 3.90
Type of housing during the course				
Family residence	35 (68.6%) *	36 (97.3%)	41 (45.6%) *	68 (86.1%)
University residence	5 (9.8%) *	0	10 (11.1%) *	3 (3.8%)
Rental apartment	11 (21.6%) *	1 (2.7%)	39 (43.3%) *	8 (10.1%)
Lunch location during the course				
Family residence	34 (66.7%) *	34 (91.9%)	37 (41.1%) *	61 (77.2%)
University residence dining room	6 (11.8%) *	0	10 (11.1%) *	3 (3.8%)
Rental apartment	9 (17.6%) *	1 (2.7%)	40 (44.4%) *	12 (15.2%)
Other place	2 (3.9%) *	2 (5.4%)	3 (3.3%) *	3 (3.8%)

Data are mean values ± SDs and percentages. Abbreviations: BMI: body mass index, SBP: systolic blood pressure, DBP: diastolic blood pressure. Normal blood pressure: systolic (120–129 mmHg) and diastolic (80–84 mmHg); prehypertensive: systolic (130–139 mmHg) and diastolic (85–89 mmHg); hypertensive: systolic (≥140–159 mmHg) and diastolic (≥90–99 mmHg). Chi-square for categorical variables; *t*-test/Welch’s *t*-test or Mann-Whitney U test for continuous variables. * *p* < 0.05 Christians vs. Muslims.

**Table 2:** Number of students who consume ultra-processed products in first and second assessments according to religion.

Ultra-Processed Products	Total (n = 257)		*p*-Value	Christians (n = 141)		*p*-Value	Muslims (n = 116)		*p*-Value
	First Assessment	Second Assessment		First Assessment	Second Assessment		First Assessment	Second Assessment	
Artificial juices	161 (62.6)	151 (58.8)	<0.001	71 (50.4)	67 (47.5)	0.046	90 (77.6)	84 (72.4)	0.014
Sugary drinks	197 (76.7)	197 (76.7)	1.000	98 (69.5)	98 (69.5)	1.000	99 (85.3)	99 (85.3)	1.000
Industrial pastries	177 (68.9)	150 (58.4)	<0.001	82 (58.2)	63 (44.7)	<0.001	95 (81.9)	87 (75.0)	0.005
Cereals	144 (56.0)	144 (56.0)	1.000	71 (50.4)	73 (51.8)	0.317	73 (62.9)	71 (61.2)	0.317
Candies	180 (70.0)	158 (61.5)	<0.001	86 (61.0)	72 (51.1)	<0.001	94 (81.0)	86 (74.1)	0.005
Chocolate	206 (80.2)	201 (78.2)	0.132	103 (73.0)	100 (70.9)	0.180	103 (88.8)	101 (87.1)	0.414
Industrial sauces	196 (76.3)	196 (76.3)	1.000	100 (70.9)	100 (70.9)	1.000	96 (82.8)	96 (82.8)	1.000
Sausages	103 (40.1)	103 (40.1)	1.000	88 (62.4)	88 (62.4)	1.000	15 (12.9)	15 (12.9)	1.000
Precooked foods	126 (49.0)	126 (49.0)	1.000	65 (46.1)	65 (46.1)	1.000	61 (52.6)	61 (52.6)	1.000

Note. Data are presented as frequency and total percentage of subjects that consume each ultra-processed product. Chi-square/Fisher’s test.

**Table 3:** Frequency of weekly consumption among Christian and Muslim students participating in the study.

Ultra-Processed Products	Total (n = 257)		*p*-Value	Christians (n = 141)		*p*-Value	Muslims (n = 116)		*p*-Value
	First Assessment	Second Assessment		First Assessment	Second Assessment		First Assessment	Second Assessment	
Artificial juices	2 (7)	2 (7)	0.180	1 (4)	0 (4)	0.779	5 (13)	6 (14)	0.138
Sugary drinks	3 (6)	3 (6)	0.001	2 (4)	2 (4)	0.027	4 (9)	5 (8)	0.007
Industrial pastries	1 (3)	1 (3)	0.001	1 (2)	0 (1)	<0.001	2 (4)	2 (5)	0.364
Cereals	1 (4)	1 (3)	<0.001	1 (3)	1 (3)	0.020	2 (4)	2 (4)	<0.001
Candies	1 (3)	1 (3)	<0.001	1 (2)	1 (2)	0.002	2 (4)	2 (4)	<0.001
Chocolate	2 (3)	2 (3)	<0.001	1 (4)	1 (3)	0.002	3 (5)	2 (4)	0.003
Industrial sauces	2 (2)	2 (2)	1.000	2 (3)	2 (3)	1.000	2 (3)	2 (3)	1.000
Sausages	0 (2)	0 (2)	<0.001	1 (2)	2 (3)	<0.001	0	0	1.000
Precooked foods	0 (1)	0 (1)	1.000	0 (1)	0 (1)	1.000	1 (1)	1 (1)	1.000

Note. Data are presented as median and interquartile range. Wilcoxon test.

**Table 4:** Belonging to the Christian or Muslim religion and its associations with the frequency of weekly consumption of ultra-processed products.

Ultra-Processed Products	n	%	OR	95% CI	OR^a^	95% CI
*Artificial juices*						
Low consumption ≤ 1	126	63.1	1			
Elevated consumption > 1	131	68.1	3.654 **	2.175–6.141	3.897 **	2.291–6.627
*Sugary drinks*						
Low consumption ≤ 1	90	45.4	1			
Elevated consumption > 1	167	77.6	2.877 **	1.663–4.977	2.923 **	1.686–5.068
*Industrial pastries*						
Low consumption ≤ 1	67	42.2	1			
Elevated consumption > 1	190	87.2	4.998 **	2.697–9.260	4.995 **	2.694–9.259
*Cereals*						
Low consumption ≤ 1	87	39.7	1			
Elevated consumption > 1	170	70.9	1.603	0.953–2.696	1.658	0.980–2.807
*Candies*						
Low consumption ≤ 1	67	39.7	1			
Elevated consumption > 1	190	85.1	3.755 **	2.072–6.804	3.724 **	2.051–6.762
*Chocolate*						
Low consumption ≤ 1	94	46.6	1			
Elevated consumption > 1	163	71.6	2.199 *	1.312–3.688	2.183 *	1.301–3.664
*Industrial sauces*						
Low consumption ≤ 1	149	65.5	1			
Elevated consumption > 1	108	48.2	0.565 *	0.341–0.937	1.762 *	1.062–2.923
*Sausages*						
Low consumption ≤ 1	169	87.1	1			
Elevated consumption > 1	88	51.8	0.138 **	0.073–0.261	0.139 **	0.074–0.262
*Precooked foods*						
Low consumption ≤ 1	202	0	1			
Elevated consumption > 1	55	100	0.926	0.508–0.688	0.962	0.522–1.774

Christians were taken as reference. Data are presented as Odds Ratios (ORs) with 95% confidence intervals (CIs) using a logistic regression model. OR^a^ adjusted for sex and religion. * *p* < 0.05; ** *p* < 0.001.

## Data Availability

The data presented in this study are available on request from the corresponding author. The data are not publicly available due to maintaining the privacy of participants.
